# SATINN: an automated neural network-based classification of testicular sections allows for high-throughput histopathology of mouse mutants

**DOI:** 10.1093/bioinformatics/btac673

**Published:** 2022-10-10

**Authors:** Ran Yang, Alexandra M Stendahl, Katinka A Vigh-Conrad, Madison Held, Ana C Lima, Donald F Conrad

**Affiliations:** Division of Genetics, Oregon National Primate Research Center, Oregon Health and Science University, Portland, OR 97006, USA; Division of Genetics, Oregon National Primate Research Center, Oregon Health and Science University, Portland, OR 97006, USA; Division of Genetics, Oregon National Primate Research Center, Oregon Health and Science University, Portland, OR 97006, USA; Division of Genetics, Oregon National Primate Research Center, Oregon Health and Science University, Portland, OR 97006, USA; Division of Genetics, Oregon National Primate Research Center, Oregon Health and Science University, Portland, OR 97006, USA; Division of Genetics, Oregon National Primate Research Center, Oregon Health and Science University, Portland, OR 97006, USA

## Abstract

**Motivation:**

The mammalian testis is a complex organ with a cellular composition that changes smoothly and cyclically in normal adults. While testis histology is already an invaluable tool for identifying and describing developmental differences in evolution and disease, methods for standardized, digital image analysis of testis are needed to expand the utility of this approach.

**Results:**

We developed SATINN (Software for Analysis of Testis Images with Neural Networks), a multi-level framework for automated analysis of multiplexed immunofluorescence images from mouse testis. This approach uses residual learning to train convolutional neural networks (CNNs) to classify nuclei from seminiferous tubules into seven distinct cell types with an accuracy of 81.7%. These cell classifications are then used in a second-level tubule CNN, which places seminiferous tubules into one of 12 distinct tubule stages with 57.3% direct accuracy and 94.9% within ±1 stage. We further describe numerous cell- and tubule-level statistics that can be derived from wild-type testis. Finally, we demonstrate how the classifiers and derived statistics can be used to rapidly and precisely describe pathology by applying our methods to image data from two mutant mouse lines. Our results demonstrate the feasibility and potential of using computer-assisted analysis for testis histology, an area poised to evolve rapidly on the back of emerging, spatially resolved genomic and proteomic technologies.

**Availability and implementation:**

The source code to reproduce the results described here and a SATINN standalone application with graphic-user interface are available from http://github.com/conradlab/SATINN.

**Supplementary information:**

Supplementary data are available at *Bioinformatics* online.

## 1 Introduction

Spermatogenesis is a developmental process in mammalian seminiferous tubules that, under normal circumstances, results in continuous sperm production. Deficiencies in this complex but essential process often result in male infertility, which is characterized by a dysfunction in sperm ejaculation or an abnormal absence of sperm in the semen and affects 2.5–12% of men (Agarwal *et al.*, 2015; World Health Organization (WHO), 2021). While key regulatory genes (Krausz and Casamonti, 2017; Mou *et al.*, 2013), chromosomal microdeletions (Ma *et al.*, 1992; Tiepolo and Zuffardi, 1976) and environmental factors (Gabrielsen and Tanrikut, 2016) have been associated with cases of male infertility, another 30–40% cases remain idiopathic (Nieschlag *et al.*, 2010), indicating that our current knowledge of the molecular machinery of spermatogenesis (Chen *et al.*, 2018; Guo *et al.*, 2017; Shami *et al.*, 2020; Suzuki *et al.*, 2019; Wang *et al.*, 2018) is far from complete.

Histology is the premier method for phenotyping spermatogenic defects. Clinically approved histopathology typically focuses on identifying only a handful of severe phenotypes, such as Sertoli cell only, germ cell maturation arrest and hypospermatogenesis (Abdullah and Bondagji, 2011; Hentrich *et al.*, 2011), which only offer insights at a coarse resolution. Systematic methods for characterizing testes in a laboratory context do exist, including traditional Johnsen scores (Johnsen, 1970) and a more comprehensive approach by McLachlan *et al.* (2007). These methods, however, are typically low-throughput due to the time-consuming nature and expertise required to manually analyze the images. Indeed, Ito and colleagues have started to address this issue by developing a method for automated classification of testis histopathology based on the Johnsen method (Ito *et al.*, 2021), highlighting the urgent need for such technologies in the field. Akin to the clinic, histology has been the basis of testis biology research and has set the foundation of understanding spermatogenesis in several key organisms, including humans (Clermont, 1966; Nihi *et al.*, 2017; Paniagua and Nistal, 1984), non-human primates (Clermont and Antar, 1973; Clermont and Leblond, 1959) and mice (Nakata, 2019; Yoshida, 2008; 2012). The classical staging system introduced by Leblond and Clermont (1952) first allowed for a systematic method to analyze testis biology from histological images by classifying cellular associations within tubules into different stages based on spermatid development. A single cross-section of a mouse testis contains ∼120 tubules that house tens of thousands of germ cells discriminated by histological markers into 20 cell types (Chiarini-Garcia and Meistrich, 2008; Drumond *et al.*, 2011) and 12 tubule stages (Ahmed and de Rooij, 2009; Oakberg, 1956; Russell *et al.*, 1990) that serve as landmarks in the cycle of spermatogenesis [reviewed in Lara *et al.* (2018)]. While the quality and quantity of testis histology has greatly improved over the last decade, computational tools capable of handling and analyzing such data are just emerging (Bell *et al.*, 2020; Dumont *et al.*, 2021; Liang *et al.*, 2022; Sziva *et al.*, 2022; Xu *et al.*, 2021).

To enable higher level analyses and to increase data processing efficiency, we aim to integrate histology with both computational image processing and machine learning. The potential of computational processing of histological images has been shown by 3D modeling of seminiferous tubules in rat (Nakata *et al.*, 2021b, c ), mouse (Nakata, 2019; Nakata *et al.*, 2015a, 2017) and Syrian hamster (Nakata *et al.*, 2021a), to better understand the physical constraints of these systems. Machine learning itself has shown useful applications in other fields, such as cancer research, where gene expression-based neural networks can distinguish between several cancer cell types (Mostavi *et al.*, 2020) and image recognition networks can detect breast cancer cells based on changes in actin filament structure (Oei *et al.*, 2019). However, apart from a few recent studies (Ito *et al.*, 2021; Liang *et al.*, 2022) including the work by Xu *et al.* (2021) using a neural network to stage Hematoxylin and Eosin (H&E)-stained tubule cross-sections, adapting learning algorithms to analyze testis histology remains largely unexplored. Here, we improve upon this idea by training a residual learning framework, or Resnet (He *et al.*, 2016), to automatically classify histological images of mouse seminiferous tubules.

Our goal in this study is to develop and assess a computational method to evaluate histopathology using automated classification of mouse seminiferous cell types and tubule stages from immunofluorescence (IF) images. To our knowledge, this report is the first of a publicly available, neural network-based classification method for IF testis images, which have unique features for the computer to learn from. Our workflow has the benefit over similar methods of making no assumptions about the composition of cells within tubules, which reduces processing times and enables functionality under non-ideal conditions, such as for meiotic-arrest mutants, which lack entire cell type populations. It also opens the door to extensive network refinement by using fluorescent markers with additional specificity, as well as downstream quantification of those markers of interest, something that would be more difficult to do using traditional immunohistochemistry stains.

In this article, we train and validate Resnet-50 convolutional neural networks (CNNs) to classify mouse seminiferous cell types and tubule stages from IF images stained with a basic set of markers. We show that we are able to computationally recapitulate the previously described meiotic-arrest phenotype of *Mlh3^−/−^* mice and use the high sensitivity of our software to make biological inferences on an undisclosed mouse mutant line that exhibits a much milder phenotype. We conclude by discussing the implications of our work to understanding the mechanical limitations of spermatogenesis as well as the research and clinical potential of combining image recognition software with the field of infertility.

## 2 Materials and methods

### 2.1 Experimental setup

Tissue was collected from sexually mature mice (>8 weeks old).

*Mlh3^−/−^* mice (The Jackson Laboratory; #018845) were bred at the Washington University in Saint Louis and processed as previously described (Jung *et al.*, 2019). Mice of the Crispy line were bred by the Ahituv laboratory at the University of California, San Francisco, and fixed testes were received in 70% ethanol. Wild-type C57BL/6J (The Jackson Laboratory; #000664) were bred in-house at the Small Laboratory Animal Unit (ONPRC/OHSU). All animal experiments were performed in compliance with the regulations of the respective host institutions.

To capture subtle image differences resulting from experimental variables, the samples used to train the neural network were processed under different conditions: (i) fixation method—perfusion or immersion; (ii) fixative type—4% paraformaldehyde (PFA) or modified Davidson’s fixative and (iii) assay—IF with or without Tyramide signal amplification and RNA fluorescence *in situ* hybridization followed by immunofluorescence. The boundaries of the seminiferous tubules were detected with anti-ACTA2 (1:100, Santa Cruz, sc-32251), the staging of tubules was performed with anti-ACRV1 (1:200, Proteintech, 14040-1-AP) and Sertoli cell nuclei marked using anti-SOX9 (1:100, Sigma-Aldrich, HPA001758). See the Supplementary Methods for a detailed description of the experiments.

### 2.2 Imaging

Whole testis sections were scanned with an Olympus VS120 slide scanner using a 40× (NA 0.95; 0.17 μm/pixel) objective and a BrightLine^®^ Sedat filter set (Semrock, DA/FI/TR/Cy5/Cy7-5X5M-B-000). Adjacent sections on the same slide without primary antibody or probe were used as negative controls to set the threshold of laser intensity during acquisition.

### 2.3 Image processing and segmentation

Images were processed autonomously using the following methods in MATLAB^®^ unless otherwise noted.

1. Intensity normalization was done using a basic top-hat filter with disk structuring element of radius 100.
2. Cell segmentation was done using the Hoechst channel in Cellpose (Stringer *et al.*, 2021) with estimated object length of 30 pixels (5 µm).
3. Tubule segmentation was done using the Actin Alpha 2 (Acta2) channel. A small amount of dilation was used to join imperfect tubule outlines, followed by whole image opening in order to remove small amounts of interstitial space (non-tubule regions). Thresholding was determined by Otsu’s method (Otsu, 1979) and the dilation factors were corrected post-segmentation.

Tubule objects were filtered for their area, between 0.5 × 10^6^ and 3 × 10^6^ pixels (roughly corresponding to tubule radii between 68 and 166 µm) and circularity >0.5 (where 0 is a straight line and 1 is a perfect circle) in order to discard incorrectly segmented or longitudinally sectioned tubules. The procedures and parameter settings described in this section were empirically determined to be suitable for our datasets.

### 2.4 CNNs and training validation

**Fig. 1.**
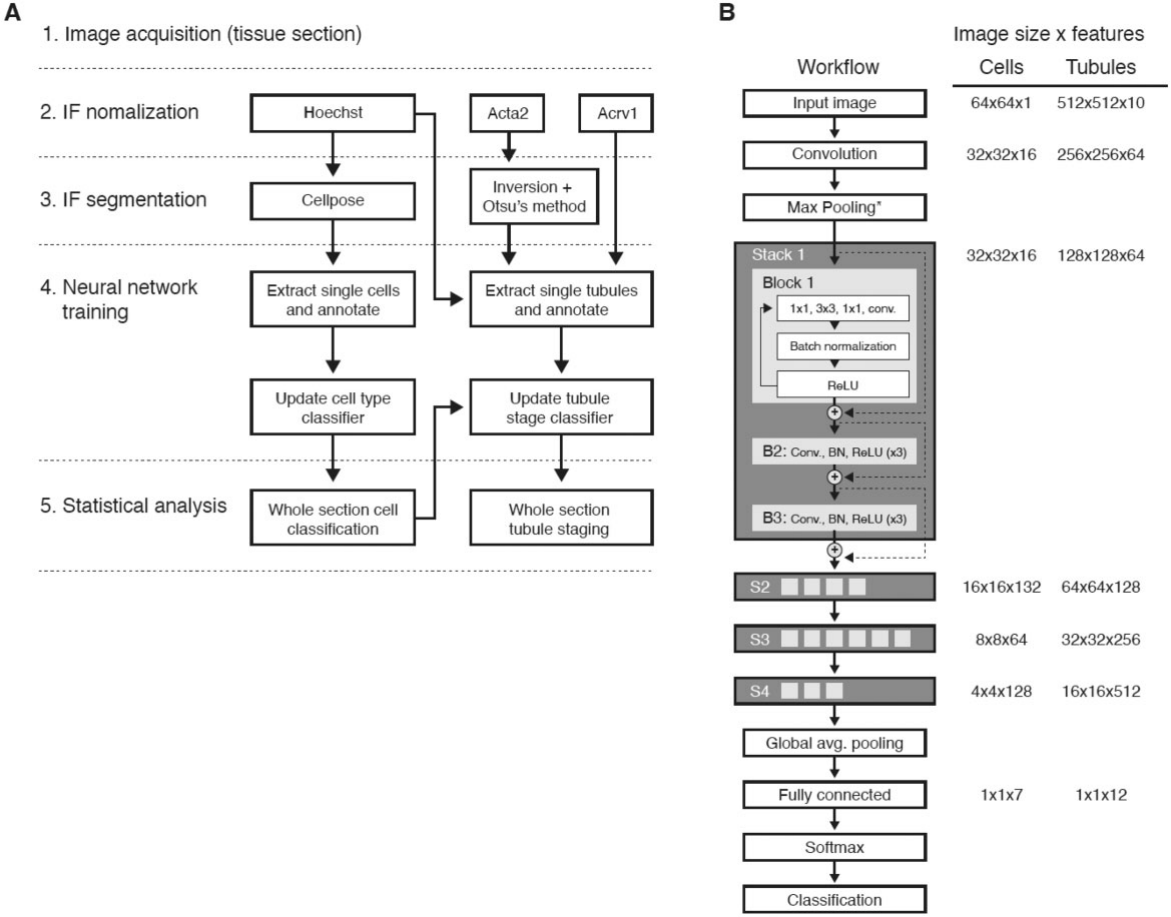
Overview of SATINN. (**A**) Image processing workflow. Raw images are acquired (see Section 2) and processed autonomously, enabling high-throughput analysis of large numbers of nuclei per image (each whole testis cross-section from mouse contains ∼120 tubules, or the order of hundreds of thousands of nuclei). (**B**) Neural network training using the Resnet-50 architecture (detailed visualization of the classifier in Step 4 of Fig. 1A). Resnets utilize a skip connection (shown here as U-shaped lines around each block) as a shortcut to learning identity functions, which greatly improves training efficiency while maintaining accuracy and enabling deeper networks. Similar overall Resnet-50 architectures are used for both cell type and tubule stage training (details on parameters are described in Supplementary Table S1)

**Fig. 2.**
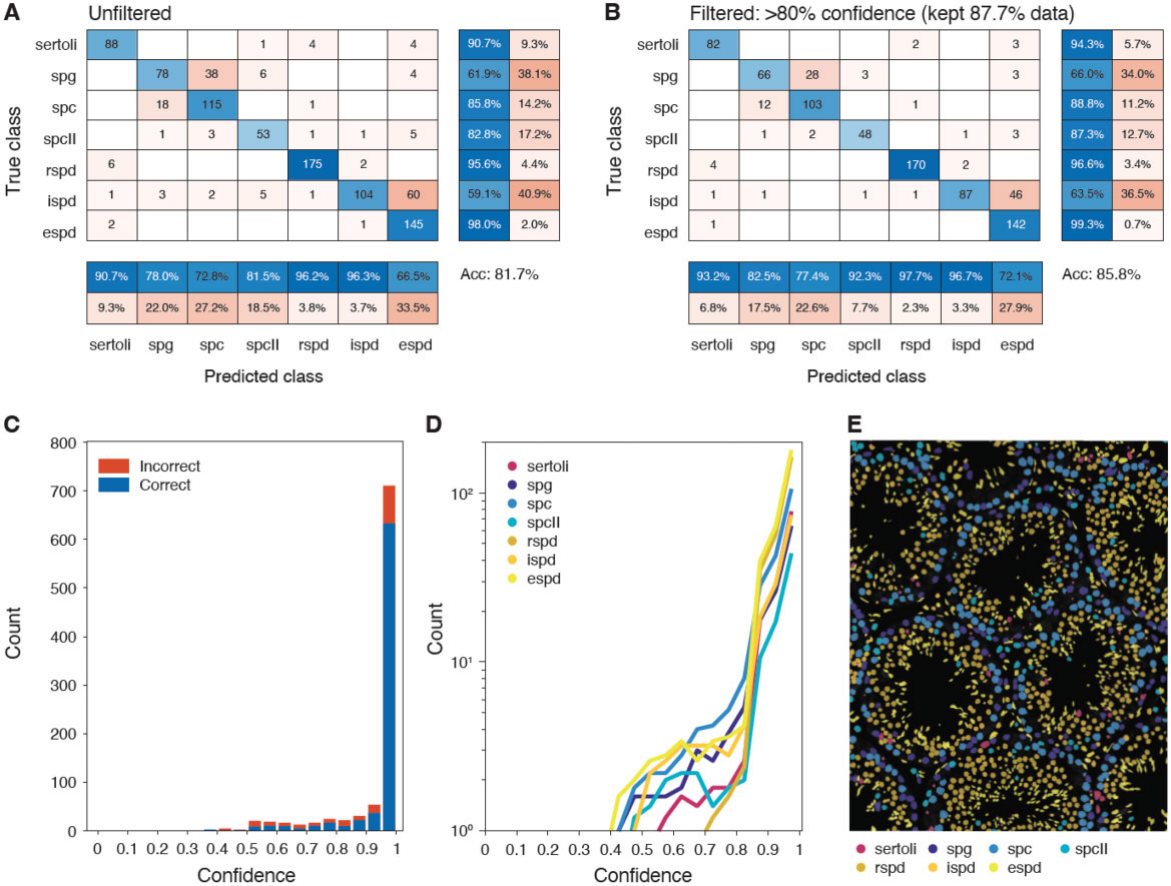
Cell type classification. We used 7032 annotated images to train the cell type classifier shown in Figure 1B to recognize seven different cell types. Another 928 images from an independent mouse were reserved for testing, and the results of this classification are shown here. (**A**) Confusion matrix. Rows indicate annotated cell classes; columns indicate CNN predictions. Bottom two rows of percentages indicate positive predictive values and false discovery rates, respectively, while right two columns of percentages indicate true positive and false positive rates, respectively. Overall accuracy of the test data was 81.7%. (**B**) High confidence confusion matrix. The same matrix as in (A), but predictions with confidence <80% were not included, retaining 87.7% of the data. The overall accuracy increases to 85.8%. (**C**) Histogram of confidence with all cells pooled into bins corresponding to their assignment confidence at 5% intervals. The highest confidence intervals also contain the highest total counts and the highest fraction of correct calls within those intervals. (**D**) Classifier confidence per cell type. All cell types have a high amount of high confidence calls. However, some developmentally adjacent cell types, such as SPG and spermatocytes, have some LCF, as expected due to attempting to discretize a continuous biological process. (**E**) Visual close-up of a tubule with cell type predictions. Each colored object represents a segmented cell; color indicates the cell type. Acc, accuracy

**Fig. 3.**
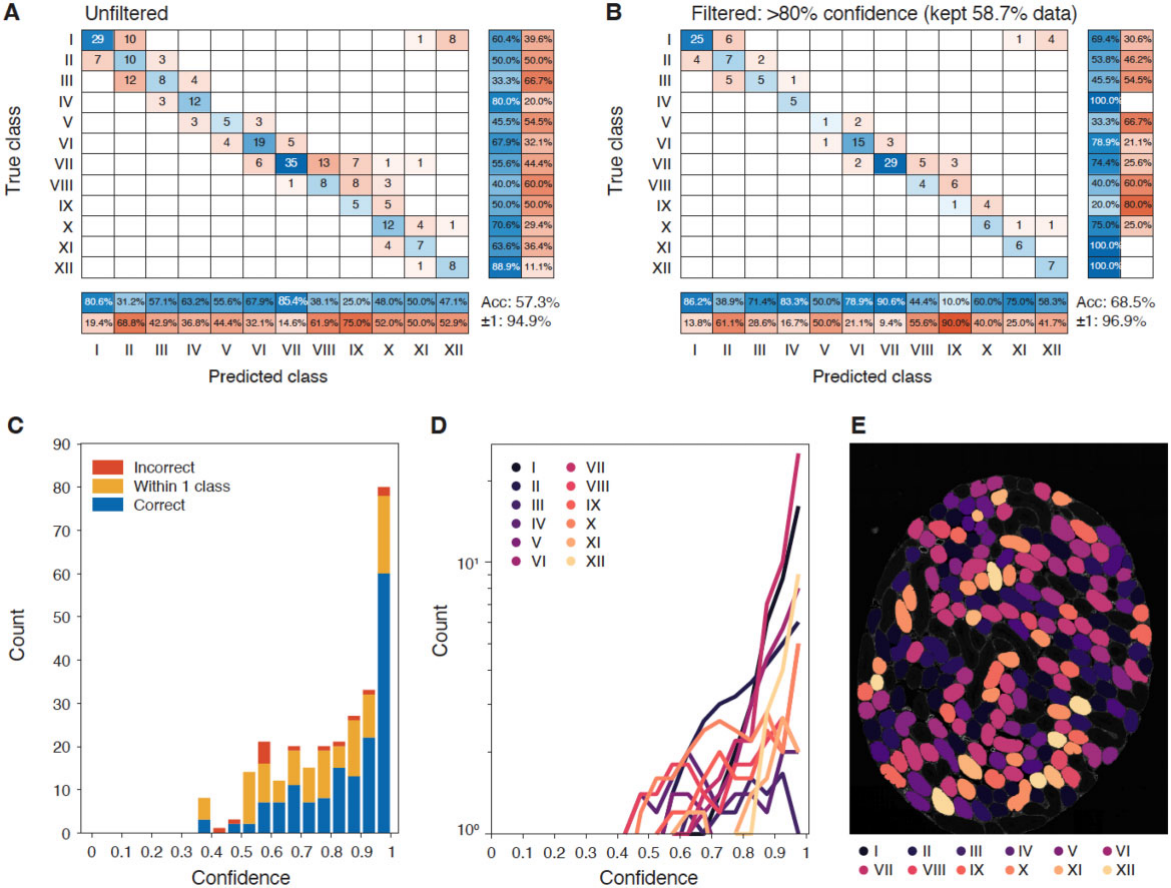
Tubule stage classification. We used 1731 annotated tubules to train the tubule stage classification CNN shown in Figure 1B to recognize 12 different tubule stages. Another 276 images from an independently annotated mouse were reserved for testing, and the results of this classification are shown here. (**A**) Confusion matrix. The overall accuracy of tubule staging is 57.3%, increased to 94.9% with one stage margin of error (e.g. Stage I can be classed as Stage II or Stage XII). (**B**) High confidence confusion matrix. (**C**) Histogram of confidence at 5% intervals with all tubule stages pooled. (**D**) Classifier confidence per tubule stage. (**E**) Visual sample of tubule segmentation and stage classification. Each object is colored by its predicted stage class. Acc, accuracy. ±1: accuracy within one adjacent stage

CNNs to classify cell type and tubule stage were designed in MATLAB^®^ with the Resnet-50 architecture (He *et al.*, 2016) (Fig. 1). The input for cells was a 64 × 64-pixel normalized Hoechst image centered on the centroid of each segmented cell, whereas for tubules, a 2000 × 2000 image downsampled to 500 × 500 containing Hoechst, Acta2, Acrosomal vesicle protein 1 (Acrv1), and cell classification channels was used instead. Objects whose CNN input image exceeded the boundaries of the source image were padded with zeros. For cell training, each image was manually annotated with its cell type. We chose to annotate intratubular cells only. The categorization of ‘intermediate spermatid’ (iSPD) refers to the developmental state between round and elongated spermatids (rSPD and eSPD) found around Stage IX. Individual cell images were sourced from 12 different testis sections to minimize bias and to acquire sufficient quantities of low-abundance cells, such as Sertoli. Likewise, for tubule training, images were manually annotated with their tubule stages, from I to XII. Tubules were sourced from the same 12 sections used to source cells, and additional care was taken to annotate low-abundance stages. Cells and tubules from a particular mouse in our datasets, MS37R1, were reserved for testing and not used to train the neural network. The results of its classification are shown in Figures 2 and 3. The custom-built code for creating these figures and analyzing the data is available at the GitHub repository (https://github.com/conradlab/SATINN).

### 2.5 Statistical distributions and tables

Tables of summary statistics for nuclei and tubules were built with the following components: image sources, classification outputs (class probabilities and calls), segmented object statistics (output of the MATLAB^®^ function *regionprops*) and computed statistics. The latter includes the following outputs of custom-built code:

1. Normalized apical-basal position (ABP) of nuclei was inferred from the distance between (i) the nucleus centroid and tubule centroid and (ii) the nucleus centroid and nearest tubule edge.
2. Relative nucleus orientation was determined by the minimum angle formed between the major axis vector of the nucleus and the vector formed between its centroid and the tubule centroid. A value close to the minimum (0°) indicates orientation along the apical-basal axis (radial) whereas one close to maximum (90°) indicates a circumferential (orthogonal) orientation.
3. Nearest neighbors were calculated for each cell (reference) connecting to the nearest cell of every other type (target). The metrics used to find each minimum include Euclidean distance and normalized apical-basal (radial) distance.

### 2.6 Quantile normalization

We used a modified version of quantile normalization that was originally described by Hicks and Irizarry (2015) in order to mitigate the impact of batch effects. Rather than quantile-normalizing a 2D matrix, we extended the design to a 3D matrix containing the following components: features (*x*-variable) are cell types; samples (*y*-variable) are the image source; and for each feature and image source, 10 000 randomly chosen observations are recorded in the *z*-direction. Each *z*-vector containing observations from a single feature-sample is sorted in ascending order, then each *xy*-frame is independently quantile-normalized using the same method as in Hicks and Irizarry (2015). *P*-values are extracted by conducting either paired *t*-tests or Mann–Whitney *U*-tests for each feature between two samples (see Supplementary Fig. S1 for visual representation).

## 3 Results

### 3.1 Neural networks classify mouse seminiferous tubules and nuclei with above 80% accuracy

To facilitate high-throughput statistical analysis of seminiferous tubules with various genetic backgrounds, we developed SATINN (Software for Analysis of Testis Images with Neural Networks) to automate cell type and tubule stage classification (overview in Fig. 1).

We acquired cross-sectional images of mouse seminiferous tubules (see Section 2 and Supplementary Methods) containing the following color channels: Hoechst (a nuclear marker), Acta2 and Acrv1. Acta2 and Acrv1 were used to assist the tubule classifier as described below. We segmented cell nuclei using Cellpose (Stringer *et al.*, 2021) and tubules using Otsu’s method (Otsu, 1979), automated object extraction, and manually annotated over 7800 cells and 2000 tubules for training and validation of the CNNs. We then built two CNNs with Resnet-50 architecture using MATLAB^®^’s Deep Learning Toolbox: a cell type classifier based on seven different cell types and a tubule classifier with 12 tubule stages.

We trained our cell type classifier using 7032 annotated images of nuclei from seven different cell types: Sertoli cells; spermatogonia (SPG); primary (SPC) and secondary (SPC-II) spermatocytes; and rSPD, iSPD and eSPD. These data originated from 70 seminiferous tubules sampled from nine testis sections from five wild-type mice. To validate our training, we reserved an additional 928 images sourced from a sixth mouse (18 tubules, two sections). We found that using only the Hoechst channel for training was sufficient to achieve a classification accuracy of 81.7% across all cell types (Fig. 2A).

Filtering out low-confidence calls (LCF, defined as below 80% confidence) increased the number of validation images being classified correctly to 85.8%, while retaining a majority (87.7%) of the data (Fig. 2B). We confirmed the robustness of thresholding in this way using two metrics: first, we observed that lower confidence calls have the highest fractions of incorrect classifications and vice versa (Fig. 2C); and second, each cell type has similar confidence distributions (Fig. 2D). Classification of nuclei from a whole wild-type section was visibly accurate (example in Fig. 2E). Though we could have increased classification accuracy by including additional cell type-specific information, such as Acrv1 to demarcate eSPDs, we prioritized simplicity of imaging requirements by minimizing the number of necessary markers and applicability of our neural network to non-wild-type tubules that may not express supplementary markers in the same way or at all.

Similar to the cell type classifier, our tubule stage classifier was trained using a set of 1731 representative images (22 testis sections from 14 mice) corresponding to various tubule stages, with an additional 276 images (two sections) from a separate mouse reserved for testing. Tubule images required Acta2 to isolate individual tubules and Acrv1 to more readily distinguish developmentally adjacent stages. We assigned classes based on the 12-stage classification system described by Oakberg (1956) and incorporated cell class predictions to further assist the tubule classifier, input as seven additional image layers of probability for each cell detected within a tubule. This resulted in a direct classification accuracy of 57.3% (Fig. 3A), though notably, the vast majority of stage classification landed within 1 stage of the true class (±1 class accuracy: 94.9%). Confidence filtering only marginally improved these values to 68.5% and 96.9%, respectively (Fig. 3B). As with the cell classifier, we ensured that the majority of calls were reasonably high confidence (Fig. 3C) and that no tubule stages in particular were more difficult to classify than any other (Fig. 3D). The result of tubule stage classification on an unannotated wild-type section is shown in Figure 3E.

### 3.2 Properties of wild-type nuclei are reproducibly measured and are resolved at the level of individual stages

**Fig. 4.**
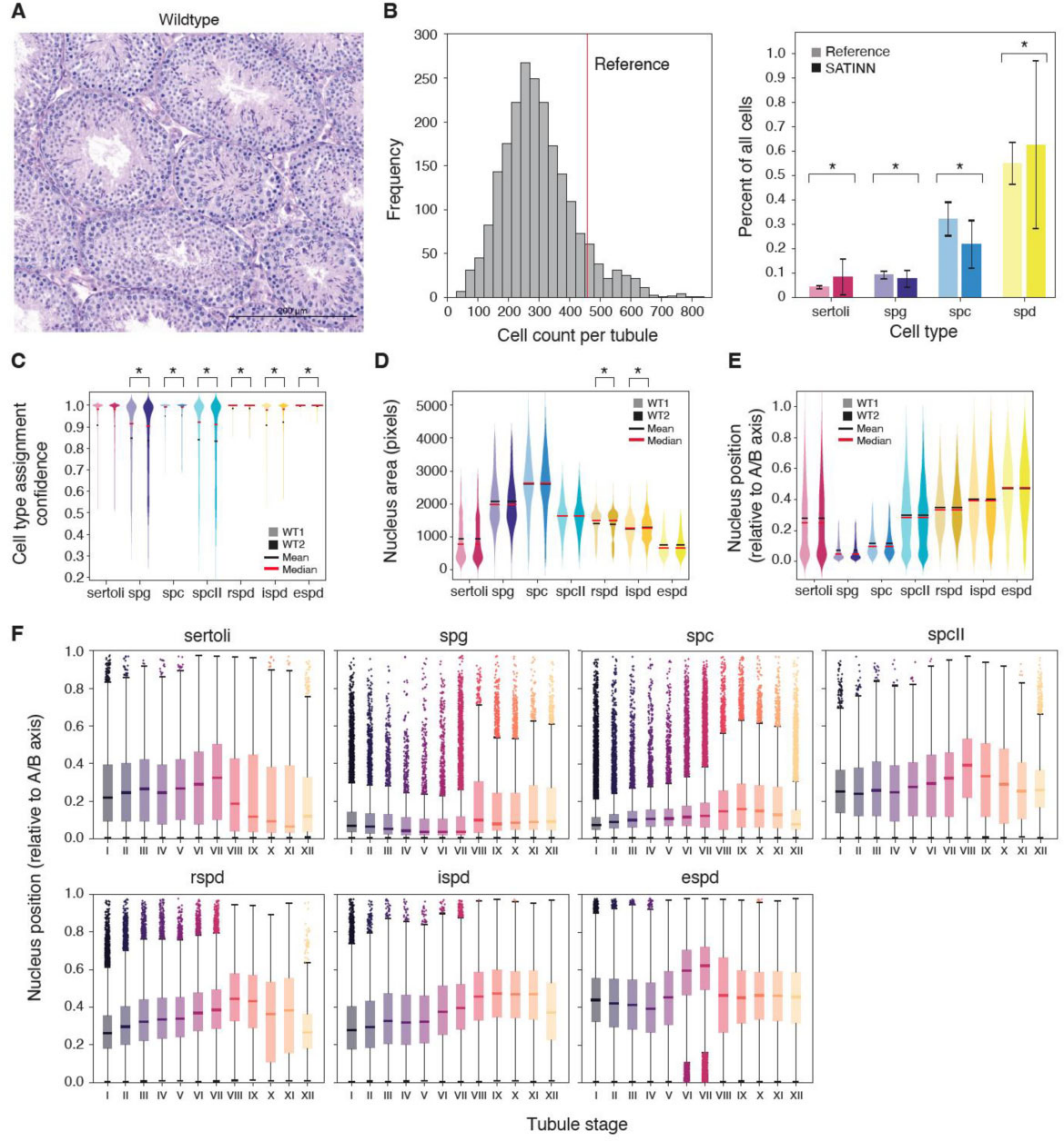
Statistical analysis of an independent wild-type dataset (not used for training or validation). (**A**) Representative brightfield image of a wild-type tubule (shown: *Mlh3^+/+^*). (**B**) Cell counts detected by our workflow, compared to Nakata *et al.* (2015b). (left) Total cell counts per tubule; (right) normalized ratios of specific cell types. Error bars SD. (**C–F**) Cross-sections of whole testes (unannotated) from five mice were imaged and split into two subgroups. Each subgroup contains an image from each mouse. Within each cell type pair (two adjacent violin plots), the left distribution originates from Subgroup 1; right from Subgroup 2. Each distribution was processed using our custom quantile normalization method (Supplementary Fig. S1). Each pair is not significant (*P *>* *0.01) unless noted. (C) Cell type assignment confidence, arranged by cell type. (D) Nuclear area in pixels. (E) ABP. This custom metric is defined as the location of a nucleus’ centroid relative to its tubule centroid and the segmented tubule edge. (F) ABP sorted by tubule stage and cell type. *P <* *10^−5^

In addition to using annotated test data to validate our neural networks, we classified unannotated wild-type tubules (representative brightfield image in Fig. 4A) to further confirm the functionality and accuracy of our classification. We first compared our CNN-derived nuclear counts with cell counts from published literature (Clermont, 1972; Oakberg, 1956; Tagelenbosch and de Rooij, 1993; Yang and Oatley, 2014); here, we show one such comparison with Nakata *et al.* (2015b). Our total segmented nuclei count per tubule was 297 ± 123 (mean±SD) sourced from over 2400 tubules (left panel on Fig. 4B). While this number is about 35% lower than the reference report due to differences in methodology (we use a nuclear marker and computerized segmentation in a fluorescence image as opposed to manual cell counting in an H&E image), the relative proportions of each cell population remain comparable (right panel on Fig. 4B).

Batch effects are a common concern with high-throughput experiments. We found subtle but significant differences among biological replicates of wild-type samples in classification confidence scores (Fig. 4C), presumably due to technical variables impossible to account for (reagent batch, operator technique, etc.). To address these batch effects, we developed a modified version of quantile normalization (Supplementary Fig. S1) that could be used with our data, and found this approach removed nearly all differences in nuclei features between two wild-type subgroups (Fig. 4D and E). The remaining features (i.e. rSPD and iSPD areas) with significant differences were due to an abundance of LCF between the two developmentally adjacent cell types, which would have been resolved post-LCF (*P *=* *0.558 and *P *=* *0.013, respectively; data and analysis not shown).

Having validated the accuracy of our cell types and tubule stages, we developed a number of quantitative measures that summarize the properties of cell nuclei within the tubules, which, to our knowledge, had not yet been rigorously quantified, including area (Fig. 4D), ABP (Fig. 4E) and relative orientation, i.e. the deviation of the object’s orientation from the apical-basal axis (Supplementary Fig. S2A–D). These measurements reflect our current knowledge from testis biology. For instance, meiotic SPC and SPCII show the largest nuclei and, together with pre-meiotic germ cells (SPG), were concentrated to the basal side of the tubule, while post-meiotic cells (spermatids) occupied apical positions. Normalized nuclear orientations did not have large magnitudes of change, though they slightly shifted toward a radial distribution (pointing toward lumen) as the cells progressed from SPCs to eSPDs (Supplementary Fig. S2E).

To better visualize spatial rearrangements in seminiferous tubules that may not be immediately apparent at first glance, we plotted ABP (Fig. 4F) and relative orientation (Supplementary Fig. S2F) with respect to tubule stage. We were able to recapitulate eSPDs moving into the lumen (more apical position) at Stages VI and VII, while having an otherwise stable ABP distribution. On the other hand, rSPDs steadily increase in ABP as spermatogenesis progresses, reflecting their apical migration as they mature. Finally, we note that Sertoli ABP drops sharply (more basal) at Stage VIII, after blood-testis barrier remodeling, while at the same time, classified SPG are shifted apically as they become committed to differentiation. Through this method, we validated many previously established observations of spermatogenesis in an unbiased way, which increased our confidence in our statistical methods and overall approach.

### 3.3 Cell type clusters are identified by nearest neighbor mapping, and spermatogenic index fluctuates with tubule stage

**Fig. 5.**
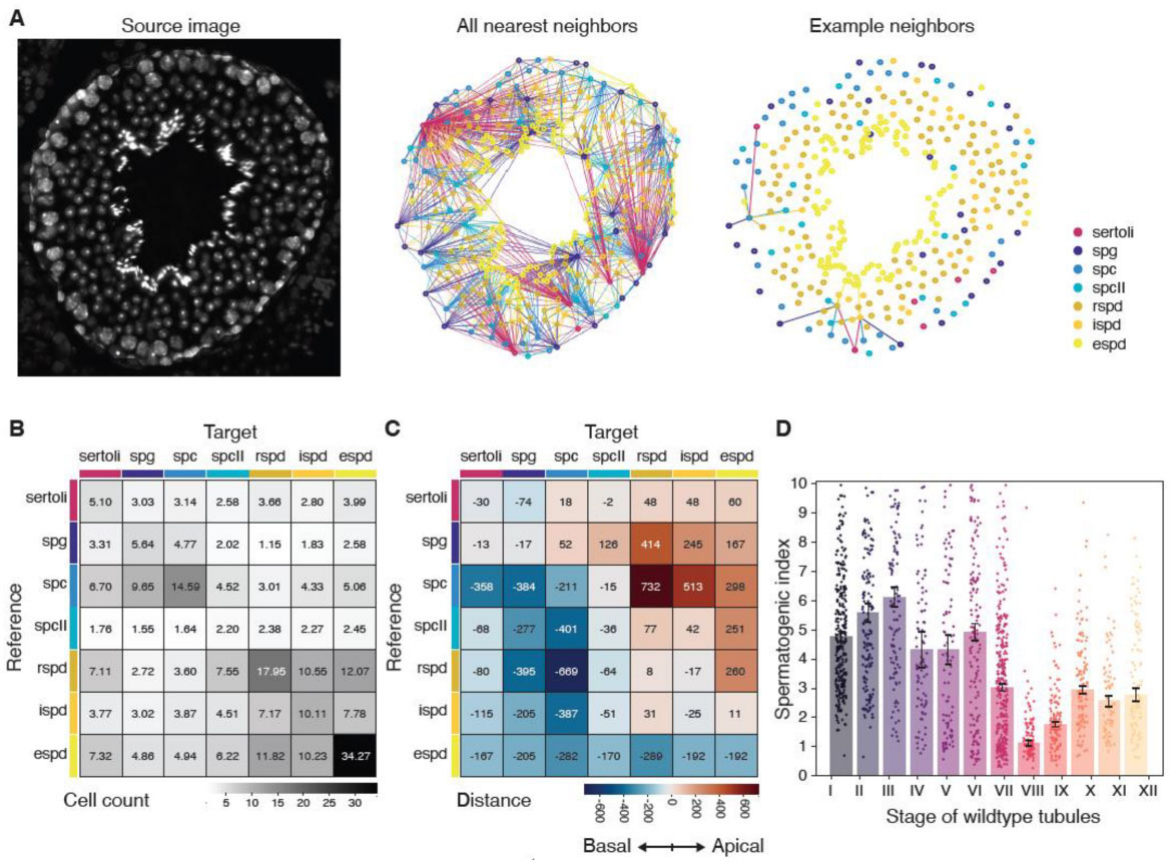
Higher order statistical analysis of an independent wild-type dataset. (**A**) Nearest neighbors analysis. (left) Sample source image of cells in a single tubule. Cells outside of the tubule boundary are dimmed. (center) Full network of nearest neighbors detected in source image. Circles represent cell centroids; their colors represent their cell type. Lines are drawn from each cell (reference) to the nearest neighbor of every cell type (targets) and are color-coded based on the target’s type. (right) Sample network from three arbitrarily chosen cells, shown for clarity. (**B**) Mean target-reference pair counts per tubule, from single nearest neighbor networks. (**C**) Mean target-reference distances. (**D**) Spermatogenic index (ratio of eSPD:SPG) of wild-type tubules sorted by annotated stage. Error bars SEM

To demonstrate higher level analytical capabilities of SATINN, we designed several statistical features, which may be useful to studying seminiferous tubule biology. We began by quantifying spatial relationships among different cell types: for each classified cell type (reference), we found the nearest neighbors of every other cell type (targets) within that tubule (example shown in Fig. 5A). We counted and normalized the cell types that correspond to the single nearest target for each reference (Fig. 5B) and found strong correlations among spermatids (rSPD, iSPD and eSPD), as well as the Sertoli-SPG-SPC block, an accurate reflection of the tissue’s architecture. We additionally analyzed radial distances between nearest neighbors, which ignores the circumferential component in order to establish apical-basal directionality between two nuclei (Fig. 5C). Consistent with our understanding of the spatial organization of these cell types, most spermatid targets averaged negative (basal) values while SPG targets averaged positive (apical). Finally, we evaluated the efficacy of spermatogenesis by calculating the spermatogenic index of each tubule, which we defined as the ratio of eSPD to SPG count. Assuming ideal meiotic conditions this value should be at least four (Hess and de Franca, 2008). When we plotted spermatogenic index in wild-type tubules with respect to stage (Fig. 5D), we found this value fluctuates depending on the tubule stage but is indeed centered around four, dropping at Stage VIII, when eSPDs are released into the lumen through spermiation.

### 3.4 Application of our workflow in histopathology quantification of a spermatogenesis-deficient mutant, Mlh3

**Fig. 6.**
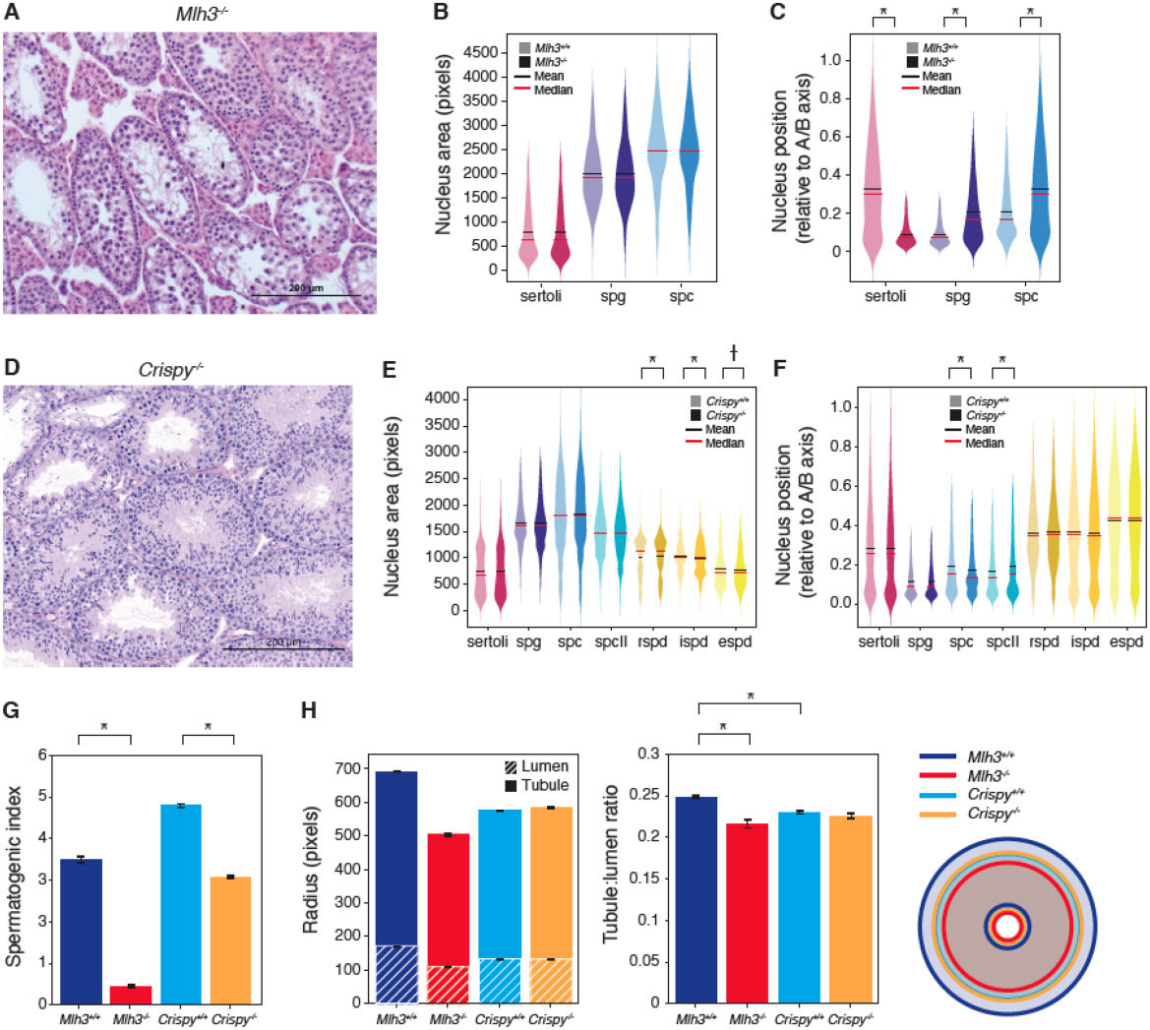
Statistical analysis of mutant cells. (**A–C**) *Mlh3^−/−^*. (A) Representative brightfield image of *Mlh3^−/−^* mutant seminiferous tubules. Three whole testis sections were derived from two mice and pooled into one subgroup (right plot in each colored pair) and plotted against *Mlh3^+/+^* Subgroup 1 (from Fig. 4, left plot in each colored pair). Each pair is not significant (*P *>* *0.01) unless noted. *Mlh3^−/−^* cells that were not classified as Sertoli, spermatocyte, or SPG were omitted for this analysis. (B) Cell area in pixels, split by cell type. (C) ABP. (**D–F**) *Crispy^−/−^*. (D) Representative brightfield image of *Crispy^−/−^* mutant seminiferous tubules. Six whole testis sections were derived from three *Crispy^−/−^* mice and pooled into one subgroup and plotted against *Crispy^+/+^* mice. Each colored pair is not significant (*P*>0.01) unless noted. (E and F) Metrics plotted are of those found in B and C, respectively. (**G**) Spermatogenic index (ratio of eSPD:SPG) sorted by genotype. *Mlh3^−/−^* values are provided pre-filtering, as the spermatogenic index would otherwise be zero by definition. (**H**) Measurements of tubule and lumen radius. (left) Bar plots indicating tubule radius (higher value) and lumen radius (lower value). (center) The calculated tubule-to-lumen ratio. (right) Visual representation of mean circularized tubules for each genotype. Error bars SEM. †*P*<10^−3^; *P<10^−5^

To test the applicability of SATINN for histopathology analysis, we used a well-characterized mutant strain with severe defects in germ cell development. Mlh3 is a DNA mismatch repair protein that has been shown to be essential for meiotic recombination during spermatogenesis (Lipkin *et al.*, 2002; Toledo *et al.*, 2019). Homozygous Mlh3-deficient (*Mlh3^−/−^*) mice are sterile, as SPCs are unable to complete meiosis, resulting in tubules with meiotic arrest and a lack of late and post-meiotic germ cells (Fig. 6A). From three *Mlh3^−/−^* cross-sections (350 tubules and 41 000 nuclei), we find that the total nuclei count in each tubule was reduced by 74.8% compared to wild-type (data not shown), which is consistent with the overall testis size reduction observed by Lipkin *et al.* (2002) and others. The primary contributing factor to the nuclei count reduction was the loss of classified spermatids (59.9% reduction of eSPD count; 89.5% of rSPD, data not shown), in agreement with classification from single-cell RNA-seq data (Jung *et al.*, 2019). To address the cell types in *Mlh3^−/−^* that were classified with an improbable cell type (i.e. spermatids and SPC-II), we filtered those classes and re-normalized the remaining distributions (i.e. of Sertoli cells, SPC and SPG, Supplementary Fig. S3A). A comparison of data management methods (removal, adjustment and no filtering) of improbable cell classes did not appreciably change the results (not shown). Our statistical analysis found little to no variation in nuclear size and orientation, as expected (Fig. 6B and Supplementary Fig. S3B, respectively). However, our computational method revealed a reversal in the spatial organization of Sertoli cells from SPGs and SPCs (Fig. 6C), as well as increasing cell density along the basal tubule edge (Supplementary Fig. S3C).

### 3.5 Detection of subtle morphological changes in crispy mutants

The hardest challenge in histopathology is the identification of minor changes undetectable by the human eye. Therefore, we tested SATINN on a mutant with a milder phenotype to calibrate its ability to detect subtle differences. To do this, we analyzed *Crispy^−/−^* mutants (Fig. 6D), a mouse line with a targeted 5 kb deletion of an evolutionarily conserved non-coding sequence that is predicted to impact spermatogenic gene expression (Okhovat *et al.*, manuscript in preparation). Analyzing six *Crispy^−/−^* cross-sections (1164 tubules and 320 000 cells), we found subtle but significant changes in the nuclear areas of most cell types (Fig. 6E) and an apical shift in nuclei location of SPC and SPCII (Fig. 6F, *P *<* *10^−5^) without disruption in other cell types.

Lastly, we performed tubule-level analysis. The spermatogenic index (Fig. 6G, ratio of eSPD: SPG counts) was close to four for both *Mlh3^+/+^* and *Crispy^+/+^* populations as expected. Despite the majority of *Crispy^−/−^* tubules appearing morphologically indistinguishable to *Crispy^+/+^*, they had a significantly lower spermatogenic index, averaging around three (*P *<* *10^−5^). This finding, which would not have been apparent in a qualitative evaluation, may provide useful insight on the functional mechanisms of this mutant. We also calculated tubule and lumen radii (Fig. 6H) for the genotypes used in this article. *Mlh3^+/+^* tubules were larger than those of *Crispy^+/+^*, which is likely a result of differences in fixation protocols for those equivalent genotypes. Nonetheless, *Mlh3^−/−^* tubules remained the smallest (*P *<* *10^−5^ when compared to *Mlh3^+/+^*), in agreement with known literature (Toledo *et al.*, 2019). On the other hand, *Crispy^−/−^* mutants are the same size as *Crispy^+/+^* (*P *>* *0.64), suggesting that the Crispy mutation does not affect tubule size. A brief calculation of the ratio of lumen-to-tubule radii revealed that these proportions remain roughly similar across all tubules, with the largest differences once again being attributed to Mlh3 mutant, though the significance values are barely below threshold (*P *=* *0.006).

## 4 Discussion

In this article, we present SATINN, a software that performs a high-throughput analysis of IF images from whole mouse testis cross-sections. We apply the image recognition capabilities of CNNs to the field of reproductive biology, resulting in automated detection and classification of thousands of nuclei into seven cell types and hundreds of tubules into 12 stages of spermatogenesis, from a single cross-section image with high accuracy. We show the benefit of collecting large amounts of data for the otherwise inaccessible exploration of fine spatial relationships between tissue structures, and how they can contribute to a better understanding of testicular biology. Importantly, we demonstrate that this software can be used to recapitulate known histopathologies of the *Mlh3^−/−^* mouse mutants and detect mild phenotypic alterations in the histology of an unpublished mouse mutant, *Crispy^−/−^*. To our knowledge, this code is the first of its kind to be publicly available and will be immediately useful to analyze IF images of mouse testis generated with the same staining schema used here (Hoechst, Acta2 and Acrv1).

The idea of using computers to assist in testis histopathology has been around for decades and is nicely reviewed by Xu *et al.* (2021). Much of this early work focused on automated identification of tubule boundaries and/or tubule classification. Most recently Xu *et al.* (2021) have begun to use neural networks for joint classification of cell types and tubule stages. This work was restricted to brightfield images, which have a more limited utility for probe multiplexing compared to fluorescence microscopy, distinguishes only three cell types and three groups of tubule stages, and does not appear to be implemented in publicly available software. Our approach has improved on these limitations through the classification and statistical analysis of more precise cell types and tubule stages, which we then applied to studying mutant morphologies. While we demonstrated SATINN’s ability to detect and quantify subtle morphological changes, it was necessary to make careful interpretations due to the limitations of image recognition software. As with all machine-learning methods that use discrete classes to categorize a continuous biological process like spermatogenesis, we expect the presence of cell type or tubule stage intermediates to arise as errors during classification. To mitigate this effect, we used a post-classification filtering based on classifier confidence (LCF), which improved the accuracy of both classifiers, and importantly, did not compromise our statistical power due to the large volume of cells and tubules that can be analyzed from each image. Additionally, due to the highly sensitive nature of CNNs, we expected the presence of batch effects and addressed them by quantile normalization (Supplementary Fig. S1). We also noticed that specific measurements could be affected by experimental conditions. Different fixatives vary in the degree of distortion produced in the tissues during fixation (Mortensen and Brown, 2003), which is reflected in our data by the significantly larger tubule radius of wild-type tubules fixed with 4% PFA (WT MS36–44) when compared to those fixed with modified Davidson’s fixative (WT 128–222; Fig. 6H). These differences could have important implications when assessing fertility of mutant mice, emphasizing the need to compare wild-type and mutant samples processed with the same experimental conditions.

Our current challenges include the difficulty of extrapolating what the neural network has learned to unknown states of pathology, including other mutant phenotypes. As we have already optimized what our classifiers can learn from our current datasets (analytics not shown), the next step would be to diversify training sources or to annotate state-specific images from other mutations, through a more streamlined training process if necessary. Training the classifier to recognize cells and cell features specifically associated with pathology is a more open-ended goal than what was attempted here but will be required if computer-assisted histopathology is to be fully competitive with human analysis. Further improvements of our current workflow to more accurately model spermatogenesis could include replacing the discrete system with a continuous or cyclical one. Additionally, tubule segmentation currently requires data from the Acta2 marker. This could potentially be overcome by using spatial cues, such as peritubular cell identification, to delimit the tubule boundaries. Integration of other molecular markers of interest would be useful in refining existing classification and enabling quantification of relevant developmental phenomena. Continued adaptation of these computational methods will ensure more reproducible and efficient image processing, along with identification of higher order features. Finally, the use of this software requires some degree of proficiency in MATLAB^®^, which is proprietary software, and will be addressed by the development of a graphic-user interface to improve accessibility.

Although the work presented here was done in mouse testes, we developed the workflow with as few assumptions as possible to enable further potential applications. A major benefit of this work is to assist fertility research in model animals, including mice, humans and non-human primates. Adaptation of SATINN in other organisms, such as humans will undoubtedly present additional challenges, such as reconciliation of the multiple tubule stages present in a single human seminiferous tubule cross-section. However, the rewards for overcoming these challenges would be greatly impactful, as the number of stages per cross-section is correlated with efficiency of spermatogenesis (Chaturvedi and Johnson, 1993) and could therefore be used as a proxy of fertility assessment. Furthermore, the ability to detect multiple proteins or mRNAs of interest is one of the hallmarks of fluorescence immunoassays. With this software, we provide the means for high-throughput analysis of molecules *in situ*, with the spatial context that only histological images can confer. One potential avenue to pursue would be to integrate automated image analysis with emerging spatial omics technologies and help bring unprecedented refinement in our ability to assess complex molecular pathways. In the long term, these improved computerized image analysis methods hold the promise of automating the analysis of testicular biopsies, a task which currently requires laborious manual evaluations performed by extensively trained experts. Prospective clinical applications of automated testis image analysis range from to the diagnosis and prognosis of assisted human reproductive technologies (Esteves *et al.*, 2018; Faes *et al.*, 2013; McLachlan *et al.*, 2007) and treatment and prevention of testicular diseases (Elsherbeny and Abdelhay, 2019).

## Supplementary Material

btac673_Supplementary_Data

## Data Availability

SATINN is freely available from https://github.com/conradlab/SATINN. Image data used in the development and testing of SATINN are available from https://figshare.com/articles/dataset/MS36R1_SEC2B_rar/19619010.
