# Supplementary material for: SATINN: an automated neural network-based classification of testicular sections allows for high-throughput histopathology of mouse mutants
Source: Bioinformatics. 2022 Oct 10;38(23):5288–98. doi: 10.1093/bioinformatics/btac673 (PMC9710558; doi:10.1093/bioinformatics/btac673)
Supplement: btac673_Supplementary_Data [file btac673_supplementary_data.docx]

Supplementary Information for Yang et al.

# Supplementary Methods

*Tissue collection and staining*

Mice were sacrificed by CO_2_ asphyxiation, testes were collected and tunica albuginea was pierced with a 31 G needle. For a subset of the samples, mice were sedated with inhaled 3% isoflurane and transcardially flushed with 0.9% saline before being perfused with 4% Paraformaldehyde [PFA; Electron Microscopy Sciences (EMS), #50-980-487] in 1X Phosphate Buffered Saline (PBS, ThermoScientific, J75889-k8). Testes were fixed overnight at 4°C in either 4% PFA or modified Davidson’s fixative (EMS, #64133-50). The next day, the tissue was either directly transferred into a 70% ethanol solution or washed in 1X PBS and dehydrated through a series of increasing ethanol concentrations up to 70% in preparation for paraffin embedding. Sections of 5 µm were used for all of the assays.

Testis were stained with hematoxylin and counter-stained with either Periodic acid-Schiff reagent (*Crispy^-/-^* testis; Thermo Scientific, #87007) following manufacturer’s recommendations, or eosin (*Mlh3^-/-^*), as previously described (Jung, et al. 2019).

*Fluorescence in situ hybridization with Immunofluorescence – FISH-IF*

FISH was performed using RNAscope® Multiplex Fluorescent Reagent Kit v2 [Advanced Cell Diagnostics (ACD), 323100] according to manufacturer’s guidelines with 15 minutes of both protease plus treatment and target antigen retrieval. Instead of counterstaining with DAPI, slides were rinsed in deionized water (diH_2_O) for immunofluorescence. Briefly, slides were incubated in blocking solution [10% normal donkey serum (Sigma-Aldrich, D9663), 1% BSA (Sigma-Aldrich, A9647-50G), 1X PBS] for 15 minutes, in primary antibody for 1 hour, in secondary antibody (1:500, Invitrogen, A21202 and A21207) for 20 minutes and in 1µg/mL Hoechst 33342 (Invitrogen, H3570) for 5 minutes. These incubations were performed at 37°C in a humid chamber in the HybEZ™ oven (HybEZ™ II Hybridization System; ACD, 321711).

*Immunofluorescence without TSA*

Tissue was deparaffinized in Xylenes and rehydrated through a series of decreasing concentrations of Ethanol. Tissue was permeabilized in 0.25% Triton X-100 (in 1X PBS), heat-induced antigen retrieval was performed in Universal Antigen Retrieval Reagent (Abcam, ab208572) and incubated in blocking solution (10% normal donkey serum, 1% BSA, 1X PBS). Primary antibodies were incubated over-night at 4°C, secondary antibodies (1:500, Invitrogen, A21202 and A21207) for 1 hour at room temperature and tissue was counterstained with 1µg/mL Hoechst 33342.

*Immunofluorescence with TSA*

Tissue was processed following the same steps as for FISH-IF but without protease treatment. Then, tissue was blocked in 1X PBS supplemented with 10% normal donkey serum and 1% BSA for 20 minutes and incubated in primary antibody for 1 hour. Two rounds of tyramide signal amplification were then performed using the Opal 4-color anti-rabbit manual IHC kit (Akoya Biosciences, NEL840001KT) with Opal 520 and Opal 570 fluorophores and anti-mouse HRP (1:500) and anti-rabbit HRP (1:500) secondary antibodies. Between rounds, RNAscope® Multiplex FL v2 HRP blocker was used to stop the amplification. There was no need to strip primary antibodies since they were raised in different species. Antibody incubations were performed for 15-20 minutes at 37°C in a humid chamber in the HybEZ™ oven and TSA for 10 minutes at room temperature.

# Supplementary Figures


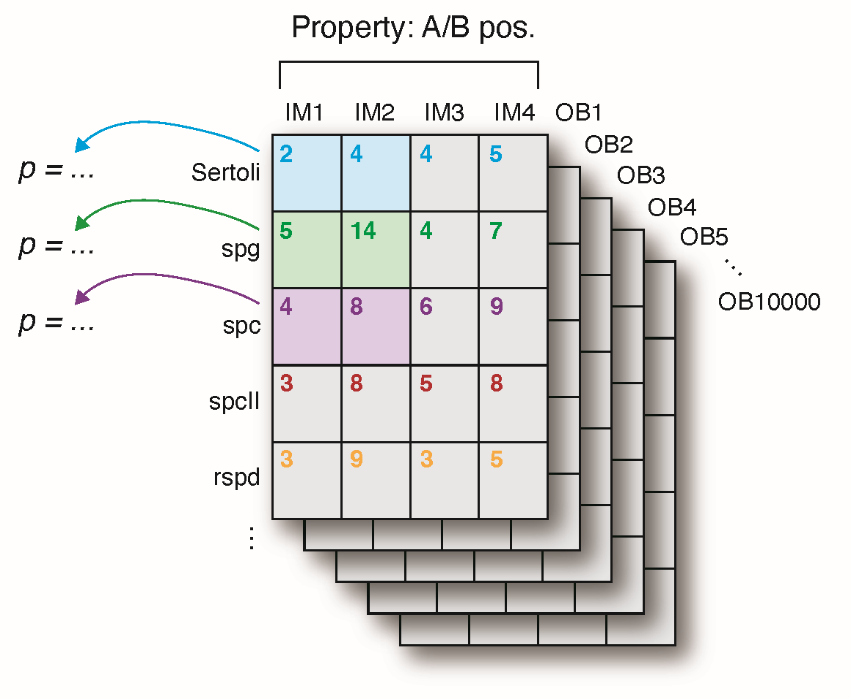


Fig. S1: Visual representation of statistical analysis using quantile-normalized data. After normalization (see methods), p-values are derived from statistical tests using observation vectors for each feature from pairs of images (each pair of colored squares represents two z-vectors from which the p-value is derived).


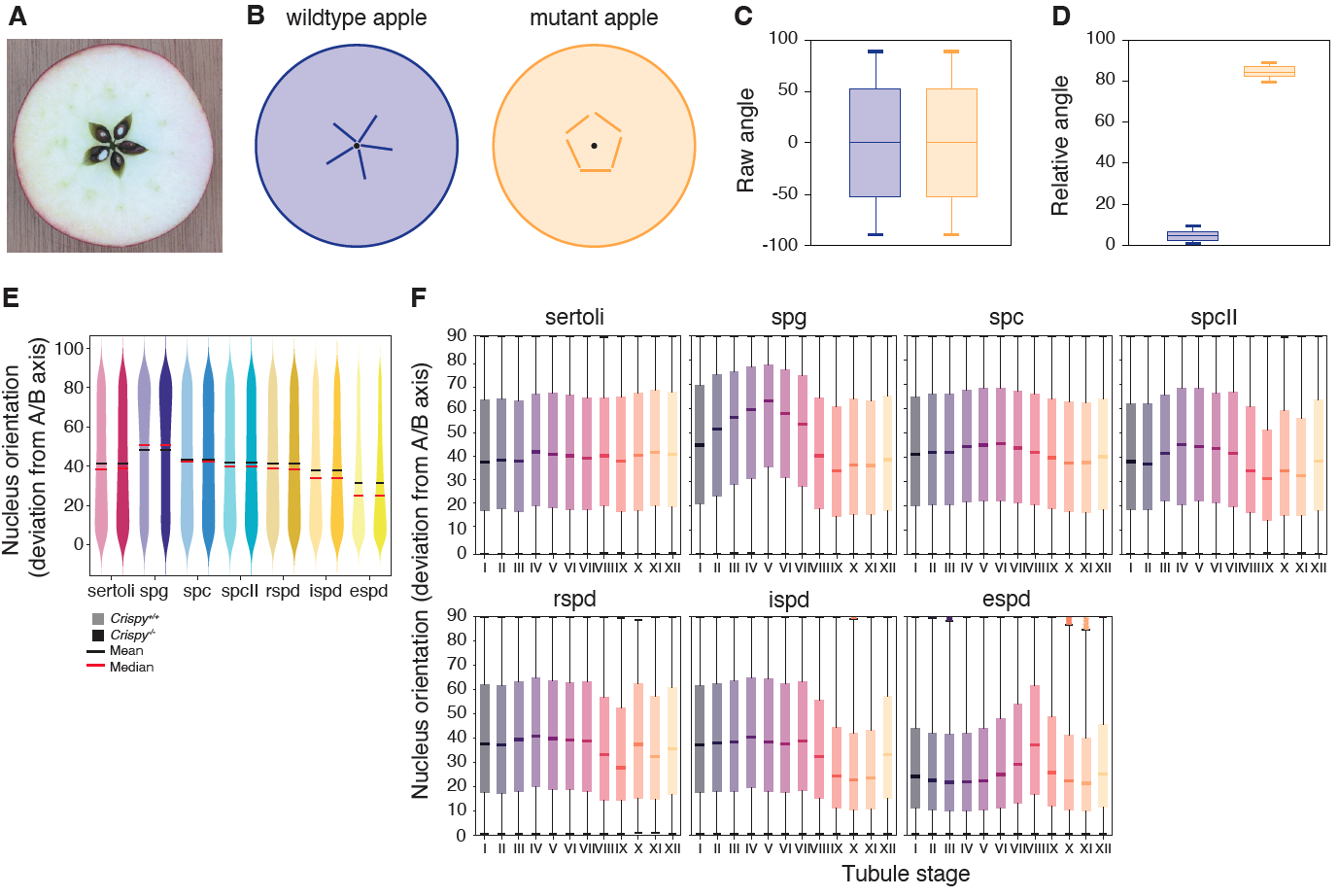


Fig. S2: Wildtype relative cell orientation plots. This statistic was developed to normalize cellular angle to a universal axis of reference for all tubules regardless of their cross-section. Cell angles are described as the offset to the radial vector, i.e. the vector between the tubule centroid and the cell centroid. The minimum possible value is 0°, corresponding to a cell whose major axis is parallel to the radial vector; the maximum possible value is 90°, indicating a cell whose major axis is orthogonal (circumferential). (A) We use an apple cross-section to illustrate the utility of this feature. (B) In the schematic for a wildtype apple (blue), the core compartments holding the seeds are oriented like a star. However, in a mutant apple (orange), suppose these vectors are now shifted so they are oriented in a circumferential direction. (C) Were we to measure the orientations of these vectors in (B) with no further context the distributions would be nearly identical. (D) However, if we were to measure their deviation from a radial vector, we can use this feature to quantify the effects of this mutation. (E-F) Actual data from wildtype tubules. (E) Orientation arranged by cell type. (F) Orientation arranged by both cell type and tubule stage.


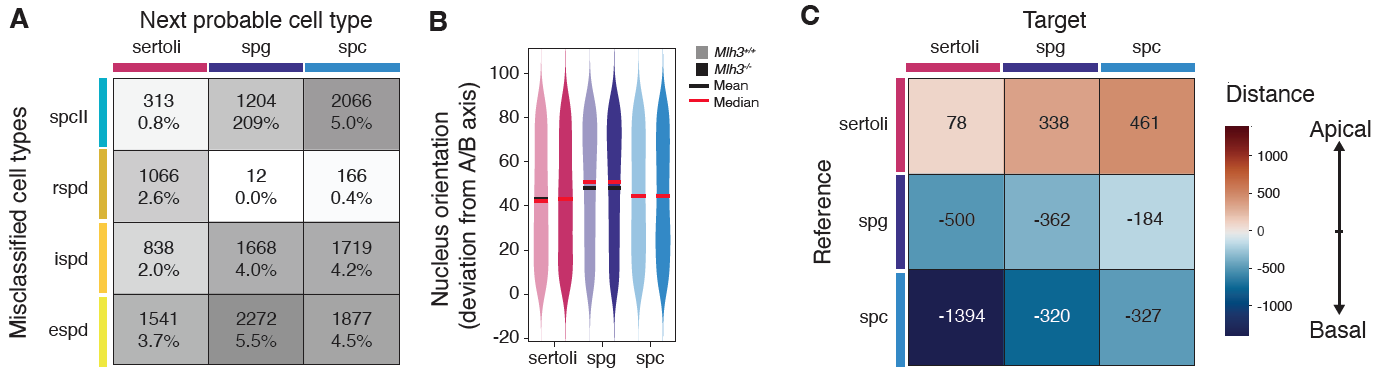


Fig. S3: Supplementary *Mlh3^-/-^* plots. (A) Classification errors in *Mlh3*^-/-^ cells have no impact on overall result whether they are omitted or changed to next probable cell type. This assessment was used to ensure our results were not biased due to our cell assignment options. (B) Relative cell orientation did not significantly change for any *Mlh3^-/-^* cell type. (C) Nearest neighbor distances for the three cell classes in *Mlh3^-/-^*. Most values are negative, indicating an increase in cell density along the basal membrane.

| Resnet parameter | Cell type classifier | Tubule stage classifier |
| --- | --- | --- |
| Initial convolution: filter size, num. filters, stride | 3x3, 16, 2 | 9x9, 32, 2 |
| Initial pooling layer | None | 3x3 max pool |
| Stack depth | 3, 4, 6, 3 | 3, 4, 6, 3 |
| Num. filters | 16, 32, 64, 128 | 64, 128, 256, 512 |
| Bottleneck residual blocks | - Batch normalization layer before addition - First convolutional layer downsamples with stride 2 | |

Table S1: Detailed neural network parameters. No initial pooling was done for the cell type classifier due to the small input size (the corresponding block marked with an asterisk in Fig. 1B was skipped).

| **Sample ID** | **Age (days)** | **Drug Treatment** | **Fixative** | **Fixation method** | **Assay** | **Stains** | **Image ID** | **Manual Annotations** |
| --- | --- | --- | --- | --- | --- | --- | --- | --- |
| MS16 | 424 | 1X PBS | mDF | immersion | IF | Hoechst, Acta2, Sox9 | InV5ms2_mDF | Cells |
| MS2 | 112 | Untreated | PFA | immersion | TSA | Hoechst, Acta2, Acrv1 | MS2-2B2-32 | Cells |
| MS2 | 112 | Untreated | PFA | immersion | FISH-IF | Hoechst, Acta2, Cldn11 / Cldn11 mRNA | MS2-2B2-24 | Cells |
| MS36 | 116 | Puromycin | PFA | perfusion | IF | Hoechst, Acta2, Acrv1 | MS36R1 | Cells; Tubules |
| MS37 | 116 | Puromycin; Cycloheximide | PFA | perfusion | IF | Hoechst, Acta2, Acrv1 | MS37R1 | Cells; Tubules |
| MS38 | 116 | DMSO | PFA | perfusion | IF | Hoechst, Acta2, Acrv1 | MS38R1 | Cells; Tubules |
| MS44 | 308 | Puromycin; Cycloheximide | PFA | perfusion | IF | Hoechst, Acta2, Acrv1 | MS44R1 | Cells; Tubules |
| MS36 | 116 | Puromycin | PFA | perfusion | IF | Hoechst, Acta2, Acrv1 | MS36R2 | Tubules |
| MS43 | 308 | Puromycin | PFA | perfusion | IF | Hoechst, Acta2, Acrv1 | MS43R1 | Tubules |
| MS43 | 308 | Puromycin | PFA | perfusion | IF | Hoechst, Acta2, Acrv1 | MS43R2 | Tubules |
| Mlh3_187 | 160 | Untreated | PFA | immersion | IF | Hoechst, Acta2, Acrv1 | Mlh3_187-1 | *N.A.* |
| Mlh3_KO192 | 160 | Untreated | PFA | immersion | IF | Hoechst, Acta2, Acrv1 | Mlh3_KO192-2 | *N.A.* |
| 128M756 | 50 | Untreated | mDF | immersion | IF | Hoechst, Acta2, Acrv1 | 128M756RTE-1 | *N.A.* |
| 130M756 | 50 | Untreated | mDF | immersion | IF | Hoechst, Acta2, Acrv1 | 130M756_RTE-1 | *N.A.* |
| 197M759 | 50 | Untreated | mDF | immersion | IF | Hoechst, Acta2, Acrv1 | 197M759RTE-1 | *N.A.* |
| 211M766 | 50 | Untreated | mDF | immersion | IF | Hoechst, Acta2, Acrv1 | 211M766_RTE-1 | *N.A.* |
| 222M704 | 50 | Untreated | mDF | immersion | IF | Hoechst, Acta2, Acrv1 | 222M704RTE-1 | *N.A.* |
| Crispy -/- 400m | 50 | Untreated | mDF | immersion | IF | Hoechst, Acta2, Acrv1 | Crispy400MB2-1 | *N.A.* |
| 83M078 | 50 | Untreated | mDF | immersion | IF | Hoechst, Acta2, Acrv1 | Crispy5-2 | *N.A.* |
| Crispy -/- 769m | 50 | Untreated | mDF | immersion | IF | Hoechst, Acta2, Acrv1 | Crispy769MB1-1 | *N.A.* |

Table S2: Details of mouse samples and experiments used for this manuscript
